# High serum levels of Dickkopf-1 are associated with a poor prognosis in prostate cancer patients

**DOI:** 10.1186/1471-2407-14-649

**Published:** 2014-09-02

**Authors:** Tilman D Rachner, Stefanie Thiele, Andy Göbel, Andrew Browne, Susanne Fuessel, Kati Erdmann, Manfred P Wirth, Michael Fröhner, Tilman Todenhöfer, Michael H Muders, Matthias Kieslinger, Martina Rauner, Lorenz C Hofbauer

**Affiliations:** Division of Endocrinology and Metabolic Bone Diseases, Department of Medicine III, TU Dresden, Fetscherstr 74, 01307 Dresden, Germany; Department of Urology, TU Dresden, Fetscherstr 74, 01307 Dresden, Germany; Department of Urology, University of Tübingen, Hoppe-Seyler-Straße 3, 72076 Tübingen, Germany; Institute of Pathology, TU Dresden, Fetscherstr 74, 01307 Dresden, Germany; Institute of Clinical Molecular Genetics and Tumor Genetics, Helmholtz Zentrum München, Marchioninistrasse 25, 81377 Munich, Germany; Center for Regenerative Therapies Dresden, TU Dresden, Fetscherstr 74, 01307 Dresden, Germany

**Keywords:** DKK-1, Prostate cancer, Prognosis

## Abstract

**Background:**

The Wnt inhibitor Dickkopf-1 (DKK-1) has been linked to the progression of malignant bone disease by impairing osteoblast activity. In addition, there is increasing data to suggest direct tumor promoting effects of DKK-1. The prognostic role of DKK-1 expression in prostate cancer remains unclear.

**Methods:**

A prostate cancer tissue microarray (n = 400) was stained for DKK-1 and DKK-1 serum levels were measured in 80 patients with prostate cancer. The independent prognostic value of DKK-1 expression was assessed using multivariate analyses.

**Results:**

DKK-1 tissue expression was significantly increased in prostate cancer compared to benign disease, but was not correlated with survival. However, high DKK-1 serum levels at the time of the diagnosis were associated with a significantly shorter overall and disease-specific survival. Multivariate analyses defined high serum levels of DKK-1 as an independent prognostic marker in prostate cancer (HR 3.73; 95%CI 1.44-9.66, p = 0.007).

**Conclusion:**

High DKK-1 serum levels are associated with a poor survival in patients with prostate cancer. In light of current clinical trials evaluating the efficacy of anti-DKK-1 antibody therapies in multiple myeloma and solid malignancies, the measurement of DKK-1 in prostate cancer may gain clinical relevance.

**Electronic supplementary material:**

The online version of this article (doi:10.1186/1471-2407-14-649) contains supplementary material, which is available to authorized users.

## Background

Prostate cancer is the most common cancer in men, and patients with advanced disease frequently develop bone metastases [1]. In bone, osteoblast functions are dependent on canonical Wnt signalling [2]. This process is controlled by Wnt inhibitors, including sclerostin and dickkopf-1 (DKK-1) [3]. Elevated levels of DKK-1 promote bone lesions in multiple myeloma and breast cancer by inhibiting osteoblast activity [4–6]. The clinical efficacy of DKK-1 inhibition is currently tested in patients with multiple myeloma [7]. The role of DKK-1 in prostate cancer, however, is less clear. DKK-1 tissue expression has been described to increase in primary prostate cancer lesions compared to normal tissue, and high DKK-1 levels within prostate cancer metastases were associated with poor survival [8]. Furthermore, in a murine model of prostate cancer, DKK-1 stimulated subcutaneous tumour growth and bone metastasis [9]. By contrast, knock-down of DKK-1 delayed the development of both soft tissue and osseous prostate cancer lesions [10]. These findings suggest that DKK-1 may have an impact on cancer biology beyond its role in malignant bone disease. Here, we assessed the role of DKK-1 expression in tissues and sera from patients with prostate cancer and evaluated its prognostic value in affected patients.

## Methods

### Tissue microarray

A prostate cancer tissue microarray (TMA) was generated from patients of clinically diagnosed and histologically confirmed prostate cancer who underwent a radical prostatectomy at the TU Dresden Medical Center of Dresden between 1996 and 2005. A total of 400 prostate cancer patients as well as 41 patients with benign prostate hyperplasia (BPH) were included. Patients with high risk prostate cancer (high Gleason score and/or lymph node metastasis) were included at a higher rate than their incidence rate for the evaluation of prognostic associations. At the time of prostatectomy and serum sampling all patients included in this study were free of clinically detectable bone lesions or other distant metastasis. TMA slides were designed to include cancer tissue from 4 different locations and 2 samples from adjacent non-tumour tissue from each patient. Slides included internal controls to ensure staining reproducibility between slides. Patient characteristics are listed in Table 1.

**Table 1:** **Patient characteristics of TMA**

Characteristics	Median (IQR) or Frequency (%)
Age at diagnosis (years)	65 (61, 68)
Preoperative PSA (ng/ml)	8.6 (5.54, 15.71)
Tumor staging (n = 400)	
pT2	178 (44.5)
pT3	154 (38.5)
pT4	68 (17.0)
Lymph node involvement	
pN0	294 (73.5)
pN1	106 (26.5)
Gleason score	
< 7	78 (19.50)
= 7	75 (18.75)
> 7	247 (61.75)

All tissue samples were obtained, stored and assessed under the same conditions as approved by the Institutional Review Board (institutional review board of the TU Dresden). Written informed consent was obtained from all patients. Histological processing was performed in the accredited Department of Pathology and conducted using a standardized procedure to assure reproducibility.

### Immunohistochemistry (IHC)

DKK-1 tissue protein levels were assessed using IHC as previously described [6]. Briefly, 2 μm-thick paraffin sections were dewaxed, rehydrated using an alcohol gradient, and heat-treated for antigen retrieval. Endogenous peroxidase activity was blocked using 0.3% H_2_O_2_/PBS for 10 min at room temperature and non-specific binding sites using the blocking buffer of the VECTASTAIN Elite ABC Kit (VECTOR Laboratories, Peterborough, UK) for 45 min. Sections were incubated with an anti-DKK-1 antibody (ab22827; Abcam, Milton, UK) overnight at 4°C. Subsequently, slides were treated with an anti-goat secondary antibody conjugated to biotin and developed utilizing avidin-conjugated HRP with diaminiobenzidine (DAKO). Specificity of the antibody has been previously validated [6]. TMAs were assessed by two experienced scientists. Staining intensity was scored as either absent (0), weak (1), moderate (2) or strong (3). Unless otherwise specified staining score is presented as the mean value of the 4 tumour or 2 adjacent normal samples from each patient.

### DKK-1 ELISA

Serum samples were available from 80 of the 400 patients included on the TMA. All serum samples were obtained after informed consent and IRB approval at the time of diagnosis, prior to prostatectomy and pharmacological treatment of the disease. Serum samples were stored under the same conditions at −80°C until use. Patient characteristics are listed in Table 2. Human DKK-1 ELISA was provided by Biomedica (Vienna, Austria) and performed according to the manufacturer’s instructions. Twenty μl of serum were incubated with 50 μl of biotinylated DKK-1 antibody for 2 hours at room temperature. Following repeated washing steps, 100 μl of conjugate were added into each well and incubated for 1 hour. After another washing step, substrate was added for 30 minutes and absorbance was measured at 450 nm with reference at 630 nm.

**Table 2:** **Clinical features of patients following division into groups according to DKK-1 serum levels (low vs. high)**

	Low DKK-1 (n = 40)		High DKK-1 (n = 40)		
	Mean ± SD	Median	Mean ± SD	Median	p-Value
DKK-1 (pmol/l)	16.18 ± 7.5	14.69	38.44 ± 7.24	38.07	<0.01
Follow up (years)	8.56 ± 2.77	8.28	8.26 ± 3.29	8.9	n.s.
Age (years)	65.03 ± 4.40	64.99	65.27 ± 5.66	64.98	n.s.
PSA (ng/ml)	14.35 ± 13.27	8.88	9.86 ± 8.98	7.21	n.s.
	n (%)		n (%)		p-Value
Tumour staging					n.s.
pT2	17 (21.3%)		18 (22.5%)		
pT3/4	23 (28.8%)		22 (27.5%)		
Gleason score					n.s.
< 7	15 (18.8%)		15 (18.8%)		
= 7	14 (17.5%)		12 (15.0%)		
> 7	11 (13.8%)		13 (16.3%)		

### Ethical approval

All human samples used in this project (serum and tissue) were obtained following informed patient consent and approval of the institutional review board of the TU Dresden (EK195092004).

### Statistical analysis

DKK-1 protein expression in prostatic tissues is presented as the mean score of all available tissue spots from each individual. Groups of two were assessed by the Mann–Whitney-U-Test, groups of three or more were assessed by ANOVA. Correlation was determined by using the Spearman's rank correlation coefficient. Serum and TMA samples were divided into two groups at the DKK-1 median and classified as high or low DKK-1. Kaplan Meier curves were assessed using the log-rank (Mantel-Cox) test. Disease-specific survival (DSS) was defined as time between surgery of the primary tumour and death of disease or time of last follow-up. For overall survival (OS), death of any cause or time of last follow-up was considered as endpoint. Univariate Cox regression was performed on each clinical covariate to examine its impact on survival. Multivariate analyses were performed in a step-wise addition of covariates significant in the univariate analyses. *P* values < 0.05 were considered statistically significant.

## Results

### DKK-1 protein levels are increased in prostate cancer tissue

**Figure 1:**
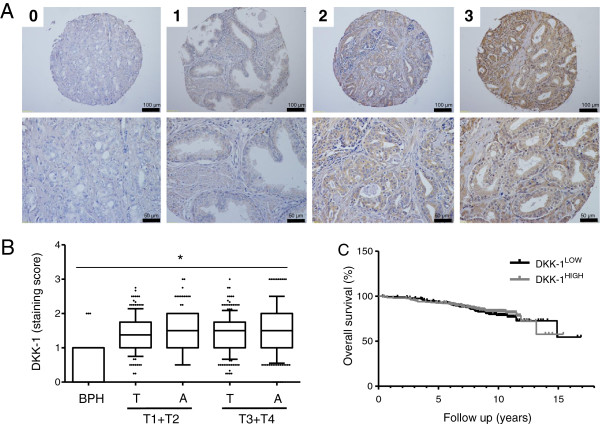
**DKK-1 tissue expression in prostate cancer. A)** The prostate TMA was immunohistochemically stained for DKK-1. Exemplary samples of each staining intensity (0–3) are shown. **B)** Distribution of DKK-1 expression in benign prostate hyperplasia (BPH), tumour tissue (T) and adjacent non-tumor tissue **(A)** is shown by boxplots. *DKK-1 tissue expression differed significantly between the different pT stages and the BPH tissues (p < 0.0001). **C)** Kaplan Meier survival analyses for PCa patients on the TMA dichotomized according to the median DKK-1 scores into high and low DKK-1 expression revealed no significant differences in overall survival (log-rank test: p = 0.27).

DKK-1 tissue expression was assessed in the prostate cancer TMA (Figure 1A). Of note, DKK-1 expression was very heterogeneous with a great variability of DKK-1 staining intensity within samples from one patient. The mean DKK-1 staining score in BPH was 0.79 ± 0.57. DKK-1 expression in prostate cancer tissue was significantly increased (mean score 1.4 ± 0.55, p < 0.0001) compared to BPH, with no differences between different tumour stages. Increased DKK-1 expression was also observed in non-tumour tissue adjacent to the tumour. There was no apparent difference between the mean DKK-1 scores of malignant and adjacent normal cells (Figure 1B + Additional file 1: Figure S1d). DKK-1 levels were significantly lower in patients with confirmed lymph node involvement (Additional file 1: Figure S1c). There was no association between DKK-1 tissue expression and clinicopathological parameters such as Gleason score, preoperative PSA serum levels and age (Additional file 1: Figure S1b + d-f). Furthermore, there was no correlation between tumour DKK-1 (high vs. low) and patient overall (OS) (Figure 1C) or disease specific survival (DSS) (data not shown).

### High DKK-1 serum levels are associated with a poor survival in prostate cancer

**Figure 2:**
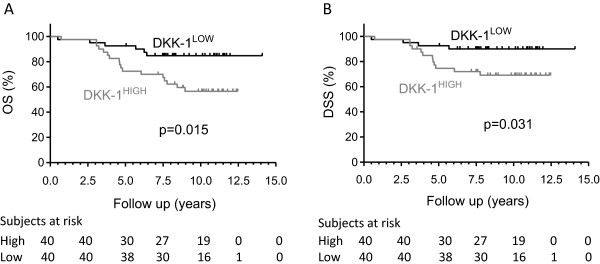
**Kaplan-Meier survival curves of prostate cancer patients showing A) DSS and B) OS in relation to their DKK-1 serum levels.** Groups were dichotomised at the median into high and low DKK-1 serum levels. Statistical assessment was performed using the log-rank (Mantel-Cox) test.

We next assessed DKK-1 serum levels in 80 prostate cancer patients that were available from patients included on the TMA. Patients were divided into two groups (high vs. low) according to the median DKK-1 serum level. There were no significant differences regarding other known patient characteristics between the two groups (Table 2). Mean DKK-1 serum levels of all patients was 27.9 ± 12.9 pmol/l. DKK-1 serum levels did not correlate with the DKK-1 tissue scores in the cancer tissue or adjacent non-tumour tissue (Additional file 1: Figure S1f). Patients with the high DKK-1 levels above the median (38.4 ± 7.2 pmol/l) were found to have a significantly shorter DSS (p = 0.031) and OS (p = 0.015) than those with low DKK-1 levels (16.2 ± 7.5 pmol/l; Figure 2). Univariate Cox regression analyses revealed significant associations between OS and pT stage, lymph node involvement, Gleason score and DKK-1 serum levels (Table 3). There was no association for age and PSA. An additional stepwise multivariate Cox proportional hazard analyses revealed that high DKK-1 serum levels were independently associated with a poor overall survival (HR 3.73; 95%CI 1.44-9.66, p = 0.007 shown in Table 3).

**Table 3:** **Uni- and multivariate Cox regression analyses for clinical characteristics and DKK-1 serum levels on overall survival in patients with prostate cancer**

Variables	Univariate analyses			Multivariate analyses		
	HR	95% CI	P value	HR	95% CI	P value
Age (years)	1.04	0.96-1.13	0.34	not included		
pT (pT3/4 vs. pT2)	11.30	2.64-48.45	0.001	8.76	1.85-41.47	0.006
pN (pN1 vs. pN0)	2.89	1.19-7.01	0.02			n.s.
Gleason	7.22	2.51-20.75	<0.001	3.34	1.10-10.29	0.036
PSA (ng/ml)	0.99	0.96-1.04	0.93	not included		
DKK-1 (high vs. low)	3.01	1.18-7.63	0.02	3.73	1.44-9.66	0.007

Abbreviations: CI = confidence interval; HR = hazard ratio.

## Discussion

High levels of DKK-1 expression in metastatic prostate cancer tissue have been previously associated with a poorer survival [8]. The role of serum DKK-1 levels in localised prostate cancer patients has not been previously investigated. In line with earlier reports in prostate cancer, we show an increased DKK-1 expression in prostate cancer tissue compared to BPH [8]. In our TMA analyses, no correlation between DKK-1 tissue expression in the primary tumour and patient survival was observed. However, DKK-1 expression within the tumour was very heterogeneous. Heterogeneity is a known finding within prostate cancer lesions [11, 12]. The heterogeneity of DKK-1 protein expression within the tumour may limit the diagnostic value of DKK-1 assessment from biopsies of the primary cancer and also explains the lack of correlation between tumour and serum DKK-1 levels. Assessment of circulating DKK-1 levels in patients at the time of diagnosis, prior to any therapy, revealed that patients with low levels of DKK-1 had a significantly better DSS and OS than those with high DKK-1 levels. A limiting factor of our study is its descriptive nature and the relatively small number of patients available for serum assessment. Larger prospective trials should be performed to further validate the findings presented here. However, there is increasing data from preclinical studies suggesting that DKK-1 may have direct effects on tumour proliferation and cell cycle. High levels of DKK-1 promoted tumour progression [9], and inhibition of DKK-1 decreased tumour burden in prostate cancer [10]. A decreased tumour burden, following DKK-1 inhibition has also been observed in multiple myeloma [13]. Interestingly, when assessing DKK-1 serum levels in patients with prostate cancer (n = 80) compared to benign prostate hyperplasia (n = 23), we did not see a significant increase (25.3 ± 6.0 vs. 27.9 ± 12.9) in DKK-1 values. This finding, together with the lacking correlation between prostate cancer DKK-1 and DKK-1 serum levels could be explained by the hypothesis that prostate cancer derived DKK-1 only modestly influences DKK-1 serum levels. If this is the case, increased DKK-1 expression in non-tumour derived tissue, as seen in our TMA, may have direct tumour promoting effects. There are an increasing number of reports that suggest different mechanisms by which DKK-1 may affect tumour biology. These anti-tumour effects appear to be, at least in part, independent of Wnt signaling and a role of p21CIP-1/WAF-1 has been suggested [10]. Recently, DKK-1 was reported to mediate tumour survival in osteosarcoma cells, via the stress response enzyme ALDH1 [14]. In addition, a negative correlation between DKK-1 serum levels and prognosis has been suggested in non-small cell lung cancer as well as cervical carcinoma [15, 16]. These observations support the role of DKK-1 as a potential tumour promoter and are fully consistent with our finding that high circulating DKK-1 levels are associated with a worse disease-specific and overall survival in prostate cancer patients. However, it remains unclear to what extent DKK-1 serum levels are tumour derived, or if high levels of circulating DKK-1 from other sites promote tumour growth and/or resistance to therapy.

## Conclusion

In conclusion, high levels of serum DKK-1 were associated with a poorer overall survival in prostate cancer patient. In light of anti-DKK-1-antibodies currently under clinical evaluation for patients with advanced multiple myeloma, these data warrant further research on the role of DKK-1 in solid malignancies, including prostate cancer.

## Electronic supplementary material

Additional file 1: Figure S1: Distribution of DKK-1 staining score across the evaluated prostate TMA (a). DKK-1 staining is separated according to Gleason score (b) and the presence of lymph node involvement (c). DKK-1 tissue expression in the tumour is shown in relation to DKK-1 expression in adjacent tissue (d), age (e) and DKK-1 serum levels. *p <0.05. (PPTX 93 KB)

## References

[1] Coleman RE (1997). Skeletal complications of malignancy. Cancer.

[2] Daoussis D, Andonopoulos AP (2011). The emerging role of Dickkopf-1 in bone biology: is it the main switch controlling bone and joint remodelling?. Semin Arthritis Rheum.

[3] Baron R, Rawadi G (2007). Targeting the Wnt/beta-catenin pathway to regulate bone formation in the adult skeleton. Endocrinology.

[4] Tian E, Zhan F, Walker R, Rasmussen E, Ma Y, Barlogie B, Shaughnessy JD (2003). The role of the Wnt-signaling antagonist DKK1 in the development of osteolytic lesions in multiple myeloma. N Engl J Med.

[5] Voorzanger-Rousselot N, Goehrig D, Journe F, Doriath V, Body JJ, Clézardin P, Garnero P (2007). Increased Dickkopf-1 expression in breast cancer bone metastases. Br J Cancer.

[6] Rachner TD, Göbel A, Thiele S, Rauner M, Benad-Mehner P, Hadji P, Bauer T, Muders MH, Baretton GB, Jakob F, Ebert R, Bornhäuser M, Schem C, Hofbauer LC (2014). Dickkopf-1 is regulated by the mevalonate pathway in breast cancer. Breast Cancer Res.

[7] Rachner TD, Göbel A, Benad-Mehner P, Hofbauer LC, Rauner M (2014). Dickkopf-1 as a mediator and novel target in malignant bone disease. Cancer Lett.

[8] Hall CL, Daignault SD, Shah RB, Pienta KJ, Keller ET (2008). Dickkopf-1 expression increases early in prostate cancer development and decreases during progression from primary tumour to metastasis. Prostate.

[9] Thudi NK, Martin CK, Murahari S, Shu ST, Lanigan LG, Werbeck JL, Keller ET, McCauley LK, Pinzone JJ, Rosol TJ (2011). Dickkopf-1 (DKK-1) stimulated prostate cancer growth and metastasis and inhibited bone formation in osteoblastic bone metastases. Prostate.

[10] Hall CL, Zhang H, Baile S, Ljungman M, Kuhstoss S, Keller ET (2010). p21CIP-1/WAF-1 induction is required to inhibit prostate cancer growth elicited by deficient expression of the Wnt inhibitor Dickkopf-1. Cancer Res.

[11] Haffner MC, Mosbruger T, Esopi DM, Fedor H, Heaphy CM, Walker DA, Adejola N, Gürel M, Hicks J, Meeker AK, Halushka MK, Simons JW, Isaacs WB, De Marzo AM, Nelson WG, Yegnasubramanian S (2013). Tracking the clonal origin of lethal prostate cancer. J Clin Invest.

[12] Ibeawuchi C, Schmidt H, Voss R, Titze U, Abbas M, Neumann J, Eltze E, Hoogland AM, Jenster G, Brandt B, Semjonow A (2013). Genome-wide investigation of multifocal and unifocal prostate cancer-are they genetically different?. Int J Mol Sci.

[13] Fulciniti M, Tassone P, Hideshima T, Vallet S, Nanjappa P, Ettenberg SA, Shen Z, Patel N, Tai YT, Chauhan D, Mitsiades C, Prabhala R, Raje N, Anderson KC, Stover DR, Munshi NC (2009). Anti-DKK1 mAb (BHQ880) as a potential therapeutic agent for multiple myeloma. Blood.

[14] Krause U, Ryan DM, Clough BH, Gregory CA (2014). An unexpected role for a Wnt-inhibitor: Dickkopf-1 triggers a novel cancer survival mechanism through modulation of aldehyde-dehydrogenase-1 activity. Cell Death Dis.

[15] Dong LL, Qu LY, Chu LY, Zhang XH, Liu YH (2014). Serum level of DKK-1 and its prognostic potential in non-small cell lung cancer. Diagn Pathol.

[16] Jiang T, Huang L, Zhang S (2013). DKK-1 in serum as a clinical and prognostic factor in patients with cervical cancer. Int J Biol Markers.

[CR17] The pre-publication history for this paper can be accessed here: http://www.biomedcentral.com/1471-2407/14/649/prepub

