# Supplementary figures and images for: High serum levels of Dickkopf-1 are associated with a poor prognosis in prostate cancer patients
Source: BMC Cancer. 2014 Sep 2;14:649. doi: 10.1186/1471-2407-14-649 (PMC4167148; doi:10.1186/1471-2407-14-649)

## Slide 1
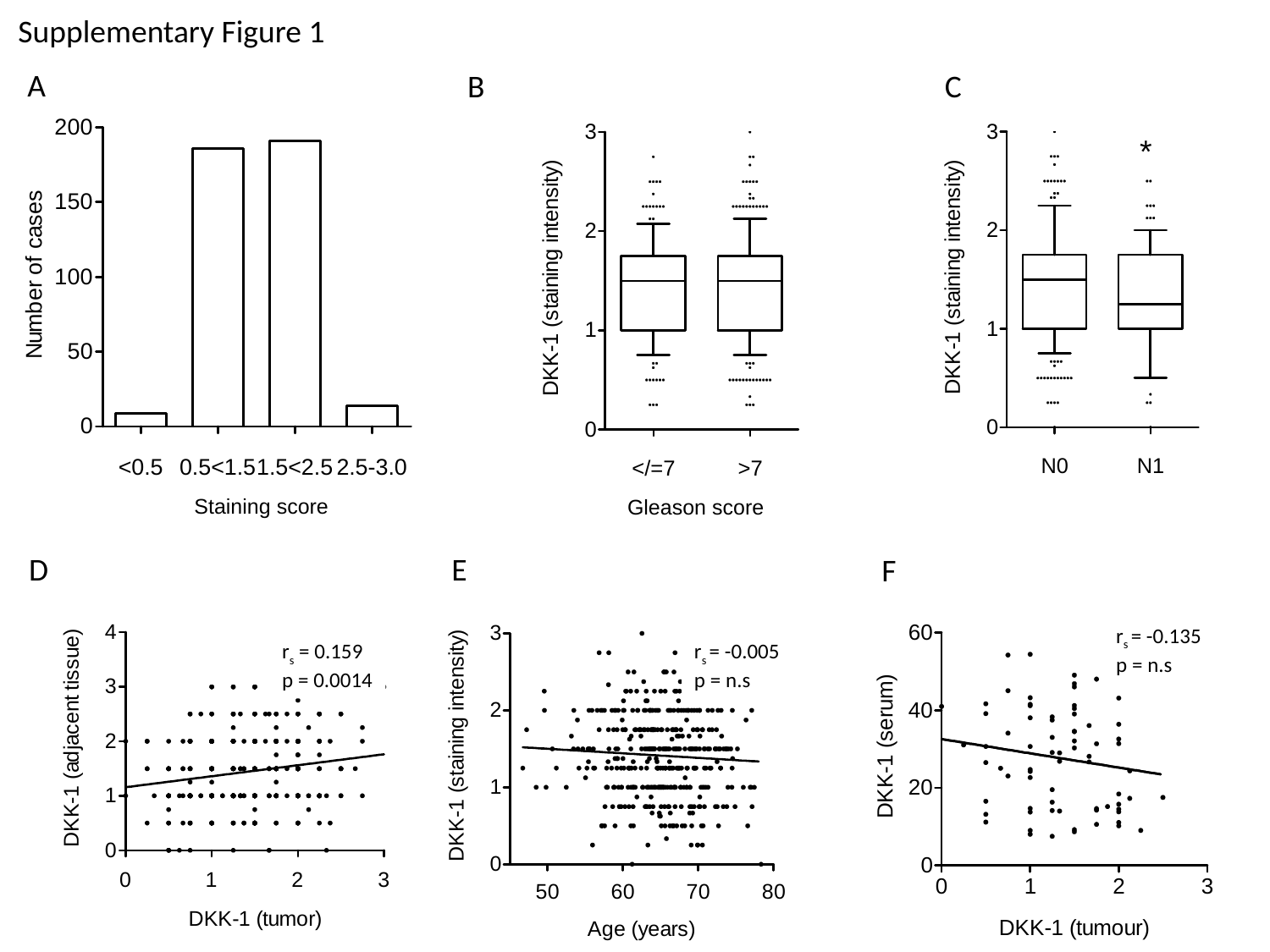

Supplementary Figure 1
A
B
C
*
Staining score
Gleason score
D
E
F
rs = -0.135
p = n.s
rs = 0.159
p = 0.0014
rs = -0.005
p = n.s

Supplement: Supplementary file 1 — Additional file 1: Figure S1: Distribution of DKK-1 staining score across the evaluated prostate TMA (a). DKK-1 staining is separated according to Gleason score (b) and the presence of lymph node involvement (c). DKK-1 tissue expression in the tumour is shown in relation to DKK-1 expression in adjacent tissue (d), age (e) and DKK-1 serum levels. *p <0.05. (PPTX 93 KB) [file 12885_2014_4843_MOESM1_ESM.pptx]
